# Assessment of In Vitro Release Testing Methods for Colloidal Drug Carriers: The Lack of Standardized Protocols

**DOI:** 10.3390/pharmaceutics16010103

**Published:** 2024-01-12

**Authors:** Laura Gómez-Lázaro, Cristina Martín-Sabroso, Juan Aparicio-Blanco, Ana Isabel Torres-Suárez

**Affiliations:** 1 Department of Pharmaceutics and Food Technology, Faculty of Pharmacy, Complutense University of Madrid, 28040 Madrid, Spain; lgomez14@ucm.es (L.G.-L.); crmartin@ucm.es (C.M.-S.); galaaaa@ucm.es (A.I.T.-S.); 2 Institute of Industrial Pharmacy, Complutense University Madrid, 28040 Madrid, Spain

**Keywords:** drug release, liposomes, microparticles, nanoparticles, sample and separate techniques, dialysis-based methods, in situ detection methods

## Abstract

Although colloidal carriers have been in the pipeline for nearly four decades, standardized methods for testing their drug-release properties remain to be established in pharmacopeias. The in vitro assessment of drug release from these colloidal carriers is one of the most important parameters in the development and quality control of drug-loaded nano- and microcarriers. This lack of standardized protocols occurs due to the difficulties encountered in separating the released drug from the encapsulated one. This review aims to compare the most frequent types of release testing methods (i.e., membrane diffusion techniques, sample and separate methods and in situ detection techniques) in terms of the advantages and disadvantages of each one and of the key parameters that influence drug release in each case.

## 1. Introduction

Drug release is part of the LADME process upon administration of a given immediate-release or extended-release dosage form. Therefore, formulation scientists seek to tailor drug release from dosage forms to specific therapeutic needs on a case-by-case basis. As a result, standardized in vitro release studies are needed as a subrogate of in vivo release kinetics for both formulation development and quality control at the industrial level [1].

Most of the currently marketed drug products are macro-sized dosage forms. The distinct pharmacopeias provide standardized procedures to determine the in vitro release profile of these dosage forms (i.e., capsules, tablets, suppositories or transdermal patches) in terms of apparatuses (basket, paddles, continuous flow), release media, agitation speed, sampling time points and tolerance thresholds of drug percentages, which must be released at given time intervals [2,3]. 

However, in contrast to macro-dosage forms, colloidal systems, such as nano- and microcarriers, lack standardized procedures to determine their in vitro drug release profiles [4]. This lack of standardized protocols is mostly due to the difficulties encountered in achieving an efficient and rapid separation of the free drug from the encapsulated one. To date, there is no compendial or regulatory standard, and the variety of testing methods makes direct comparison among the release profiles from different colloidal systems difficult [5]. 

On the one hand, colloidal systems with sizes above 400 nm can be useful for the development of prolonged-release systems over several weeks. As it also occurs with macro-dosage forms, the establishement of pharmacopeia tolerance release studies for extended-release colloidal carriers could contribute to gain insight into their in vivo performance by using in vitro drug release studies. This highlights the importance of defining standardized protocols to evaluate extended-release properties with an adequate technique and to control batch-to-batch variability [6,7].

On the other hand, nanomedicines with a high surface area usually increase the therapeutic index of drug substances mostly through targeted delivery [8,9], where drug release should occur subsequently to distribution to the target site. This drug release can be stimuli-triggered to ultimately be site-specific [10,11]. These stimuli-responsive nanocarriers have shown the ability to trigger drug release using both internal (pH [12] and ionic strength [13]) or external (temperature [14], ultrasound [15], magnetism [16] and light [17]) stimuli. Finding standardized protocols to evaluate drug release in stimuli-responsive systems is required to evaluate the extent to which drug release is stimulus-dependent or not and ultimately prevent drug release from occurring before reaching the biophase. 

The fact that standardized methods are not available for colloidal systems leads to an unpredictable therapeutic outcome and thus a high risk of failure in clinical trials, which greatly accounts for their limited clinical translation. Despite the promising benefits of these formulations at a preclinical level, only a few colloidal systems are approved for drug delivery in comparison with macro-sized dosage forms [18,19].

Therefore, this review aims to compile the existing methods to determine in vitro release from colloidal carriers, including their limitations and applicability, so that it serves as a guideline for formulation scientists to select the most suitable technique to determine the drug release profile in each case. The main body of this review is divided into three distinct types of techniques (sample and separate methods, dialysis-based methods and in situ detection systems) and their variants to evaluate in vitro drug release. Each technique serves to evaluate different colloidal systems listed from micro- to nanosized. In turn, within each type of system, distinct encapsulated drugs are reviewed, focusing on their solubility and molecular weight.

## 2. Sample and Separate Methods

**Figure 1:**
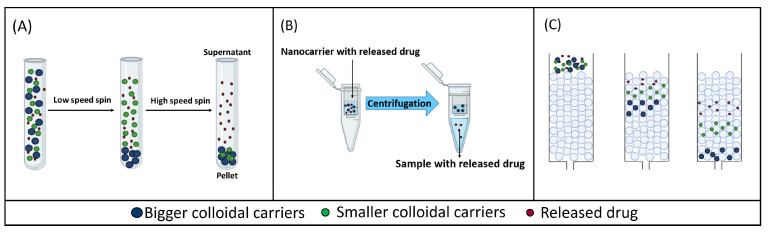
Schematic representation of different sample and separate methods. (**A**) Centrifugation/ultracentrifugation. (**B**) Centrifugal ultrafiltration. (**C**) Size exclusion chromatography.

In sample and separate methods, the drug-loaded colloidal carriers are first dispersed in the release medium and incubated at a physiological temperature under stirring. Then, at different sampling time points, aliquots are withdrawn. These aliquots include both free and encapsulated drugs, so these samples must go through a separation technique to isolate the released drug from the encapsulated fraction at each sampling time point [20]. In contrast to macro-dosage forms, conventional filtration is not a useful technique for colloidal systems since it does not achieve an efficient separation between the released and the encapsulated drug because colloidal systems can permeate through the filters to some extent [21]. Alternatively, adequate separation techniques for colloids include centrifugation/ultracentrifugation [22], centrifugal ultrafiltration [23] or size exclusion chromatography [24] (Figure 1).

Since the carriers need to be diluted in the release medium to achieve sink conditions [25], depending on drug solubility and the quantification limit of the analytical method, the total volume of the medium can range from 1 [26] to 900 [27] mL. Based on the volume of the release medium used, in vitro release studies have been performed in vials, when small volumes are used (<10 mL) [25,26,27], or USP I, and II apparatuses, when higher volumes are used (10–900 mL) [28,29]. 

### 2.1. Centrifugation and Ultracentrifugation

These separation methods are based on using high centrifugal forces over a short period of time to separate the released drug from the carrier-bound drug, as the latter forms a sediment in the sample tube (Figure 1A). Bigger-sized colloidal systems, such as microcarriers, can be separated from the released drug using low relative centrifugal force (RCF). However, nano-sized carriers require an increase in the RCF to be separated from the surrounding medium in which the released drug remains dispersed [30,31,32]. 

The main difference between centrifugation and ultracentrifugation is the centrifugation speed that is achieved: in centrifugation, the RCF reached is a maximum of 100,000× *g*, whereas in ultracentrifugation, this is higher than 100,000× *g* [21]. These techniques have been used to study the release from different colloidal carriers such as microparticles, liposomes and nanoparticles, as shown in Table 1. 

#### 2.1.1. Microparticles 

Drug release from microparticles of both macromolecules and small molecule drugs has been evaluated using centrifugation. Sanchez et al. developed two types of microparticles loaded with interferon-alpha (IFN-α, 22.1 kDa): PLGA reservoir-like microparticles containing IFN-α co-encapsulated with poloxamer with an average size of 10 µm and IFN-α loaded PLGA/poloxamer microspheres in two different sizes (10 and 40 µm). In their study, centrifugation (2000× *g*; 15 min) was used to determine the in vitro release of this protein from both types of microparticles [33]. Phosphate buffer saline (PBS) pH 7.4 added with 0.02% *w/v* Tween 80 was used as the release medium. Tween 80 was added to the release media to prevent the particles from clumping and to improve their wettability. At each sampling time, supernatants were removed after centrifugation, and the sediment was redispersed in fresh release medium to maintain sink conditions.

In a subsequent study, Brauner et al. investigated the release of trimethoprim (290.3 Da) from 1–9 µm sized PLGA 503H (molecular weight of 24–38 kDa) microparticles and PLGA 2300 (molecular weight of 2–2.5 kDa) microparticles [34]. To assess in vitro drug release, they used centrifugation (20,817× *g*; 15 min), and they performed this study using artificial urine (pH 5) at 37 °C as a release medium to simulate the intended site of action of trimethoprim, i.e., the urinary tract. After 8 h, a double amount of trimethoprim was released from 503H PLGA microparticles compared with 2300 PLGA microparticles, whereas total drug release occurred after 8 and 9 days, respectively. The authors concluded that these centrifugation conditions allow for different release profiles from these microparticles to be detected when the type of PLGA is modified, although the results did not correlate with the molecular weights of the distinct PLGAs, with a higher burst release for the microparticles made of the PLGA with the higher molecular weight.

In both cases, Sanchez et al. and Brauner et al. used release studies with centrifugation to guide the optimization of the microparticle formulation of a given drug amount to achieve the most suitable release profile for each therapeutic purpose.

#### 2.1.2. Liposomes

Focusing on lipophilic drugs, in vitro curcumin (368 Da) release from liposomes has been studied with centrifugation [35]. These liposomes were made with soybean phospholipids (SPCs) and hydrogenated soybean phospholipids (HSPCs). Varying the SPC/HSPC ratio (10:0, 7:3, 5:5, 3:7 and 0:10), systems with an average size of 84, 93, 183, 220 and 146 nm, respectively, were obtained. In vitro release of curcumin was studied using centrifugation at 15,000× *g* for 30 min in an in vitro simulated gastrointestinal tract model, which mimicked three distinct digestion points: saliva fluid (composed of NaCl, KCl and mucin, pH 6.8), gastric fluid (containing NaCl, HCl and pepsin, pH 1.5) and intestinal fluid (K2HPO4, NaCl, bile salts and pancreatin, pH 7). These three simulated digestive steps lasted 10 min, 2 h and 2 h, respectively. Using these centrifugation conditions, it was possible to evidence different trends in curcumin release profiles based on the integrity of the liposomes in each simulated medium. In the case of the simulated saliva, the integrity of all liposomal formulations was maintained because of the absence of specific enzymes that could disrupt phospholipid bilayers and the short processing time, which accounted for the low curcumin release during this stage. In the gastric fluid, liposomes with higher amounts of SPC showed a higher release of curcumin due to their lower stability. However, in simulated intestinal digestion, a marked increase in curcumin release was observed for all liposomal formulations due to the hydrolysis of the phospholipids of the liposomes by lipolytic enzymes such as pancreatin and the emulsifying effects of bile salts on liposomal membranes. In simulated intestinal fluid, 10:0 SPC/HSPC liposomes, which were less stable, released most of the remaining curcumin in a burst manner, whereas 0:10 SPC/HSPC liposomes, which were more stable, revealed a sustained release profile.

#### 2.1.3. Polymeric Nanoparticles

On the one hand, regarding hydrophilic macromolecules, Cai et al. studied the release of the protein bovine serum albumin (BSA) (66 kDa) from 40–1000 nm sized chitosan/tripolyphosphate (TPP) nanoparticles using centrifugation [36]. For this purpose, centrifugation conditions were first optimized in terms of centrifugation speed and time to achieve maximum separation efficiency. To this end, they used ultracentrifugation with a range from 2.1 × 10^4^ to 3.0 × 10^5^ RCF and a range of centrifugation time from 30 to 90 min. When the centrifugation time was fixed at 30 min and the centrifugal force was increased from 2.1 × 10^4^ to 3.0 × 10^5^ g, the percentage of particles recovered in the sediment increased from 60 to 85%. These results were supported by the percentage of encapsulated BSA recovered in the sediment, which increased from 26 to 64% with the increase in centrifugation force. With regards to centrifugation time, when RCF was fixed at 3.0 × 10^5^ and centrifugal time was increased from 15 min to 2 h, the percentage of particles recovered in the sediment increased from 82 to 95%. An analogous trend was also observed for the percentage of encapsulated BSA recovered in the sediment in this case. This previous analysis concluded that low centrifugation forces and/or short centrifugation times failed to ensure a complete separation between the particles and the supernatant, which could lead to an overestimation of actual release rates by considering non-sedimented encapsulated drugs as released drugs. As a result, both very strong centrifugal forces and prolonged centrifugation times were required to achieve complete separation of the particles from the supernatant. After this previous process to fix centrifugation conditions, the authors evaluated the influence of ionic strength and solvent replacement frequency in the release profile. First, to explore how the ionic strength of the release medium can influence the measured release rates, they performed a BSA release experiment using 5 wt% trehalose solutions in the presence and absence of NaCl as the release medium. Over the first hour, the BSA release from chitosan/TPP particles placed in 150 mM NaCl was three times higher than that achieved without NaCl. Moreover, the percentage of BSA released in one week was two times higher in the case of the release medium with 150 mM of NaCl compared with the release medium in the absence of NaCl. Since the pH of the release medium exceeded the protein’s isoelectric point, the BSA was negatively charged at the physiological pH. Therefore, the increased ionic strength caused dissociation of the ionic interactions between BSA and the chitosan/TPP particles with electric-field screening, leading to nearly complete BSA release. Secondly, in the study where aliquots were withdrawn and replaced with an equal volume of release medium, the authors explored the influence of solvent replacement frequency. They compared the release profiles obtained using 1, 4, 12 and 24 h intervals between solvent replacement. A faster BSA release was obtained when shorter periods of time (i.e., 1 and 4 h) were used compared with longer time intervals (i.e., 12 and 24 h). This was due to the fact that sink conditions were not maintained within the 12 and 24 h interval time and the accumulation of BSA in the release medium limited further release. These results showed that frequent medium replacement can be a solution when working with sink conditions is difficult.

Along the same line, another study evaluated the release of BSA from five different particle sub-groups of PLGA nanoparticles (100–250 nm; 250–500 nm; 500–750 nm; 750–1000 nm; >1000 nm) [37]. The study was carried out with centrifugation (14,000× *g*; 15 min) using PBS supplemented with 0.01% Tween 80 as a release medium. These fixed centrifugation conditions allowed for the influence of the average diameter of the nanoparticles in the release profile to be established: particles with the smallest average diameter (100–250 nm) showed a three times higher burst release than those with the largest average diameter (>1000 nm), resulting in a shorter total release period for the smallest ones compared with the biggest ones.

On the other hand, related to hydrophilic small-molecule drugs, Brauner et al. investigated the release of trimethoprim (290.3 Da) from both 200–400 nm PLGA 503H nanoparticles and PLGA 2300 nanoparticles [34]. They used artificial urine (pH 5) as a release medium and centrifugation (20,817× *g*; 30 min; 4 °C) as the separation technique. In comparison with the previously described centrifugation conditions used to investigate trimethoprim release from microparticles by the same authors, in the case of nanoparticles, the time required for centrifugation was higher (30 min vs. 15 min) to achieve nanoparticle sedimentation at the same centrifugation force. Moreover, compared with microparticles, the release of the antibiotic from nanoparticles was increased for both PLGA types: after 2 h, 60% of trimethoprim was released, and complete release was reached after 24 h, with no significant differences in release profiles between the polymers.

With regard to small lipophilic drugs, Abouelmagd et al. studied the release of paclitaxel (853 Da) from 161 nm sized PLGA nanoparticles [38]. The centrifugation conditions used were 10,000 rpm and 10 min, and three different types of release medium were used: PBS (a release medium in which the solubility limit of paclitaxel was exceeded for the amount of drug added), PBS with 0.2% of Tween 80 (close to the solubility) or PBS with 50% of FBS (sink conditions). These different release media were used to prove if these centrifugation conditions were suitable to establish different release profiles when the release medium is modified to maintain sink conditions. In FBS/PBS, nanoparticles released 98.7 ± 11.0% of the paclitaxel payload after 72 h. In Tween/PBS, nanoparticles released 83.9 ± 1.3% in 72 h, whereas in PBS, the release was relatively small, reaching a cumulative release of 34.2 ± 6.4% in 72 h. These results showed that in the experiments where sink conditions are ensured, a complete release of paclitaxel is achieved in three days and that the reported centrifugation conditions were a suitable separation method.

#### 2.1.4. Highlights of Centrifugation and Ultracentrifugation as Separation Methods

One of the advantages of centrifugation is that, compared with the other techniques used to assess in vitro drug release, it is the least resource-consuming technique because of the possibility of separating the different components of the suspension just by adjusting centrifugation parameters: lower RCF and shorter periods of time for bigger carriers as microparticles and higher RCF and longer periods of time for smaller carriers as nanoparticles and liposomes. This separation process occurs because the particles are denser than the aqueous release medium, so centrifugation can sediment the carriers while keeping the released drug in the supernatant [33,34]. Although valid for both micro- and nanocarriers, this technique seems to be more suitable for bigger-sized carriers than for smaller ones. Centrifugation has also been used both for macromolecules (IFN-α) and small-molecule drugs (trimethoprim). 

Focusing on its disadvantages, weak centrifugal forces might not ensure complete separation of the carriers from the supernatant containing the released drug. This fact was claimed by Cai et. al, who concluded that ultracentrifugation was required to achieve efficient separation [36]. Therefore, it is necessary to verify beforehand if the RCF allows an efficient separation of the released drug from the carrier.

However, the centrifugation force must also ensure that the integrity of carriers is maintained, as in this technique, an overestimation of drug release can occur because the high centrifugation force needed to achieve an efficient separation can alter the carrier structure [30]. Moreover, in those cases where the entire volume of the sample is centrifuged, subsequent resuspension is required to carry on with the studies [33]. If particle disruption occurs because of the centrifugation procedure, it is not possible to recover them to continue with the release study, and it would require the use of independent sample replicates for each sample timepoint. Alternatively, if only small aliquots are withdrawn and centrifuged at each sampling point, there is always some remaining medium that is not centrifuged to continue the release study.

Moreover, since no other mechanism apart from centrifugal force contributes to separation, it normally must be conducted over significant periods of time, which can represent a significant added period over the fixed sampling points in which release can also continue. Therefore, this technique is not useful for early time points if release is not prevented during the centrifugation time by cooling down the samples [39]. 

In conclusion, centrifugation and ultracentrifugation are used to study the release from carriers bigger than 100 nm and do not seem to be suitable for smaller carriers. The centrifugation conditions must ensure a complete separation between the released drug and the encapsulated drug. A more efficient separation is achieved with a greater difference in size between the colloidal system and the encapsulated drug.

### 2.2. Centrifugal Ultrafiltration 

Another method for separating the released drug from the carrier-bound drug is the centrifugal ultrafiltration technique, which is a sample and separate technique that can separate the free drug from the colloidal suspension by applying relatively low centrifugal forces (8000–10,000× *g*) over a short period of time. In this technique, samples are placed in the upper part of a centrifugal ultrafiltration unit, which includes a semi-permeable membrane with a specific molecular weight cut-off (MWCO). The MWCO should be selected on a case-by-case basis so that carriers are retained over the size threshold, whereas the free drug freely permeates across the membrane to be ultimately recovered in the centrifugate after centrifugation (Figure 1B) [40]. This method has been used to study the release from small nanocarriers (50–400 nm), such as liposomes and nanoparticles, as shown in Table 2.

#### 2.2.1. Liposomes

Fugit et al. studied the release of topotecan (421.44 Da) from 100 nm sized pegylated liposomes [23]. Drug release was monitored with centrifugal ultrafiltration: each aliquot was ultrafiltered using a centrifugal filter device with 30 kDa MWCO membranes and centrifuged at 14,000 rpm for 10 min. This centrifugal ultrafiltration method served to evidence differences in release profiles by varying the medium pH conditions. The maximum amount of drug released was achieved using a pH of 3.35–4.10 (about 80% after 6 h); however, when the pH of the release medium was 5.10, approximately 80% of the topotecan was retained inside the liposomes over the same period of time.

Analogously, the release of ciprofloxacin (331.3 Da) from a liposomal formulation composed of 80 to 90 nm unilamellar vesicles was carried out. HEPES-buffered saline (HBS: 20 mM HEPES, 145 mM NaCl pH 7.4) was used as a release medium. The amount of drug released and encapsulated was measured with centrifugal filtration. Before carrying out the release study, the authors validated the method to establish the most suitable conditions to achieve complete separation of the free drug from the encapsulated one. To this end, they used two types of membranes (10 and 30 kDa MWCO) and varied the centrifugation time (5, 10, 12, 15 and 18 min) at a constant 8100× *g* centrifugation force. With regard to the MWCO of the membrane, no differences were observed between both membranes. Moreover, when they evaluated the influence of centrifugation time, they observed that the most suitable period was 10 min because the recovery of ciprofloxacin was close to 95% when centrifugal ultrafiltration was carried out with free ciprofloxacin. In contrast, with shorter periods of time, the recovery was slightly lower, and with longer periods of time, no higher recovery of ciprofloxacin was achieved [41,42].

#### 2.2.2. Micelles

Ponta et al. prepared 100 nm sized micellar formulations loaded with doxorubicin (543.5 Da) [43]. These micelles were prepared from block copolymers of poly(ethylene-glycol) (PEG) and poly(aspartate) with hydrazide-conjugated doxorubicin. The hydrazide linker was selected to be labile at acid pH values. The release of the drug was studied under non-sink conditions in an acid medium using potassium biphthalate sodium hydroxide buffer solution (pH 5.0) as well as in neutral conditions using potassium phosphate monobasic buffer solution (pH 7.4). Centrifugal ultrafiltration was carried out with centrifugal filter membranes of 10 kDa MWCO at 14,000 rpm for 10 min. The pH effect on drug release was observed after 72 h, with more doxorubicin being released in acid conditions than in neutral ones, which means that this centrifugal ultrafiltration technique was able to show different release profiles depending on the pH.

#### 2.2.3. Polymeric Nanoparticles

Drug release of hydrophilic molecules from polymeric nanoparticles has been evaluated using centrifugal ultrafiltration. In this way, Rodrigues et al. studied the release of primaquine (259.34 Da) from 150–200 nm sized poly (D, L-lactide) (PLA) nanoparticles [44]. To this end, nanoparticles were diluted in different volumes of PBS pH 7.4 at 37 °C (10–1000 mL). Aliquots from the release medium were processed with centrifugal filter membranes of 3 kDa MWCO at 1000× *g* for 5 min. This method was sensitive enough to show differences in the release profiles when different dilutions of the colloidal carrier were carried out to maintain sink conditions. Specifically, when the dilution factor was increased from 1/10 to 1/500, an increase in the release of primaquine was observed (30.6 ± 1.2% to 69.6 ± 2.9%) because the system was more saturated with a lower dilution, whereas significant differences were not observed between the dilution factors of 1/500 and 1/1000 (69.6 ± 2.9% and 70.9 ± 3.5%, respectively).

On the other hand, the release of lipophilic drugs from polymeric nanoparticles has also been studied. In this sense, Weng et al. studied the release of three lipophilic small-molecule drugs: itraconazole (705.63 Da), cholecalciferol (384.63 Da) and flurbiprofen (244.26 Da), from d-α-tocopheryl polyethylene glycol 1000 succinate nanoparticles with sizes of 90–95 nm, 40–45 nm and 25–35 nm, respectively [47]. To carry out the release study, they set up the centrifugal parameters at 1000× *g* for 5 min and used 30 kDa membrane filters. Distinct release media were selected to maintain sink conditions in each case. A total of 5 or 10 mL of a suspension of nanoparticles with 2.5 mg drug were added into a USP dissolution apparatus II with 900 mL of the specific release medium for each drug at 37 °C. For centrifugal ultrafiltration, 5 mL samples were withdrawn and put into the centrifugal unit. Different release profiles were obtained: after 100 min, only 5% of cholecalciferol and 20% of itraconazole were released, whereas 99% of flurbiprofen was released.

#### 2.2.4. Lipid Nanoparticles

Focusing on hydrophilic molecules loaded into lipid nanoparticles, Barbosa et al. studied the release of dibucaine (343.46 Da) from 200 nm sized nanoparticles prepared using two different excipients: myristyl myristate and cetyl palmitate [48]. A centrifugal ultrafiltration membrane of 10 kDa was utilized, using phosphate buffer (pH 7.4) as the release medium and fixing the centrifugation at 4100× *g* for 20 min. This method showed slight differences in the release profiles when varying the excipients of the formulation. For both types of nanoparticles, the release profile began with a burst release in the first hour: for myristyl myristate nanoparticles 40% of dibucaine was released, while 50% of dibucaine was released from cetyl palmitate nanoparticles. After that, in the following 2 days, a sustained release profile took place with an additional 10% released over the initial burst release for both nanoparticles.

#### 2.2.5. Highlights of Centrifugal Ultrafiltration as a Separation Method 

This technique has mainly been used for small-molecule drugs (and not for macromolecules) encapsulated in nanocarriers (and not in microcarriers). Theoretically, with an adequate selection of the MWCO for the membrane, this separation technique is deemed to be suitable for any drug or carrier size. However, for macromolecules and microcarriers, centrifugation/ultracentrifugation techniques are preferred over centrifugal ultrafiltration because adequate separation is achieved, and they are more economical since they do not require filtration units. 

One of the advantages of centrifugal ultrafiltration is that, due to the presence of a semi-permeable membrane, which enables the free drug to diffuse through it but not the encapsulated one, the time and centrifugation force required for the separation process can be low. As previously reviewed, centrifugation does not reach values above 8000–10,000× *g*, and it is carried out over a short period of time, usually 5–10 min. For this reason, the integrity of the particles is less compromised. However, as Kyle et al. and Cipolla et al. indicated, there are some cases in which, due to the small size of the carrier, longer periods of time are required to achieve an efficient separation. In these cases, the centrifugation process should be carried out under refrigerated conditions (4 °C) to prevent the drug from being released during the centrifugation process itself [41,48,49]. 

Regarding the disadvantages of this technique, as a consequence of using a membrane to achieve efficient separation, this method cannot be used for dispersions with high particle content due to the sedimentation of particles that ultimately clogs the filter and does not allow the released drug to pass freely, which results in inadequate separation of the samples [21]. 

### 2.3. Size Exclusion Chromatography 

Size exclusion chromatography (SEC) can also be used for the separation of nanocarriers in suspension from the released drug. In SEC, a stationary phase, which is composed of a polymer or silica-based column, and a variable mobile phase are used [50]. The packing material of SEC columns contains small and rigid pores, so SEC separates molecules according to their size, or more specifically, to their hydrodynamic volume. The larger particles in a sample will elute earlier than smaller molecules because they are trapped in fewer pores of the column packing material (Figure 1C) [50,51]. 

This technique is more used to determine encapsulation efficiency [52,53] rather than to assess drug release. For example, Han et al. used the same Sephadex G-50 chromatography column to separate paclitaxel-loaded cationic solid lipid nanoparticles and doxorubicin-loaded PEGylated liposomes from free drugs and determine the encapsulation efficiency [24]. Paclitaxel is a small lipophilic drug, whereas doxorubicin is a small hydrophilic drug, which means that size exclusion chromatography can serve to separate both water and non-water-soluble drugs.

#### Highlights of Size Exclusion Chromatography as Separation Method

This technique can separate the released drug from the colloidal carrier according to their hydrodynamic volume with no centrifugation force being required. As a result, no damage to the particle is expected to occur. 

The pore size of the packing material should be carefully selected because if the molecular weight of the released drug is much smaller than the pore size, it will be retained in the stationary phase and its quantification will not be possible.

The most significant disadvantage of the SEC analysis is the adsorption of nanoparticles to the column packing material. This adsorption can also prevent the results from being quantitative [50]. 

## 3. Dialysis-Based Methods 

Of all the methods used to assess drug release from nanosized carriers, dialysis methods are the most versatile and popular. In these methods, the separation of the released drug from the nanocarriers is achieved using a dialysis membrane with an appropriate MWCO [53]. This semi-permeable membrane separates two chambers: a donor compartment and an acceptor compartment. 

The donor compartment is where the colloidal suspension is placed, while the acceptor compartment is where the release medium is placed and where the released drug appears. Samples taken from the acceptor compartment at periodic intervals can be directly measured without any further separation steps as, in principle, only released drug molecules pass across the membrane, while nanocarrier-bound drugs are unable to penetrate through it. 

Here, we describe methods to achieve an efficient separation based on dialysis membranes, including dialysis bags and diffusion cells, which include Franz cells and side-by-side diffusion cells. In these techniques, there are some parameters that influence drug release, including the MWCO of the semi-permeable membrane, agitation conditions, release media and temperature. It has been suggested that the dialysis membrane for drug release testing should have an MWCO that is at least 100 times higher than the molecular weight of the released drug to ensure that the released drug can freely pass through but not the encapsulated one [54]. 

Agitation should be used to minimize the unstirred water layer effect, which means the formation of a drug-saturated area around the membrane, leading to a violation of sink conditions since the drug is not distributed homogenously in the compartment. Therefore, it should be used in both the acceptor and donor compartments, but this is only possible in side-by-side diffusion cells, whereas in dialysis bags and Franz diffusion cells, stirring is only feasible in the acceptor compartment. 

Regarding the release medium, the sink conditions of the drug in the release medium must be ensured to enable its diffusion across the dialysis membrane. Furthermore, in these release studies, the temperature should be maintained to mimic physiological conditions. 

### 3.1. Dialysis Bag 

**Figure 2:**
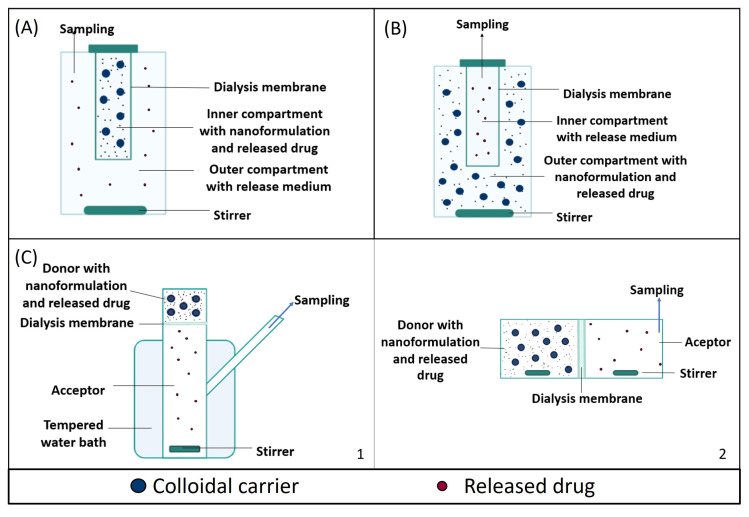
Schematic representation of different dialysis-based methods: (**A**) dialysis bag, (**B**) reverse dialysis and (**C**) diffusion cell (a Franz diffusion cell (**C1**) and side-by-side diffusion cells (**C2**)).

The dialysis bag technique is widely used to evaluate drug release from nanosized carriers, as summarized in Table 3. In this technique, the sample is placed into a dialysis container, which includes a dialysis membrane and acts as the donor compartment. The principles of filtration and diffusion guide this technique as a free drug, with a molecular weight below the MWCO, passes through the membrane while nanoparticles are retained (Figure 2A). In general, the volume enclosed in the inner compartment (1–10 mL) is significantly smaller than that of the outer medium (typically around 40–500 mL) to ensure drug diffusion under sink conditions [55]. 

#### 3.1.1. Microparticles

Shi et al. prepared 0.5–6 µm sized PLGA microparticles containing ciprofloxacin (331.3 Da) and magnetic nanoparticles [56]. In their study, 1 mL of microparticle suspension was placed in a dialysis bag (12–14 kDa MWCO) with PBS as the release medium. The release study was performed in a thermostatic shaker at 37 °C and 100 rpm, both in the presence and in the absence of an oscillating magnetic field to trigger the release of ciprofloxacin from the microparticles. This dialysis bag technique showed that when a magnetic field was applied, a higher amount of ciprofloxacin was released. This occurred because of the poration caused in the microparticles by the mechanical or thermal forces generated by the magnetic field.

#### 3.1.2. Liposomes 

As for liposomes, there are some studies that investigated the release of small hydrophilic drugs using dialysis bags. Chi et al. studied the glutathione-responsive release from 165 nm sized cationic liposomes loaded with doxorubicin (543.2 Da) [57]. For this purpose, the liposomal suspension was placed into a dialysis bag (10 KDa MWCO), kept at 37 °C in a shaker incubator and dialyzed against 30 mL of release medium. Two types of release medium were used: PBS (pH 7.4) containing 50% fetal bovine serum (FBS) and PBS with different concentrations of glutathione (GSH) (0, 20 µM and 10 mM) to mimic the hypoxic conditions of a tumor. After sampling, the volume was replaced with an equal aliquot of fresh medium. This technique allowed for discerning doxorubicin release under physiological (PBS + FBS) and tumor (PBS + GSH) conditions. Under physiological conditions, 35% of doxorubicin was released in 48 h, and a similar amount of doxorubicin was released when 20 µM GSH was added. However, 10 mM of GSH triggered a two times higher burst release of doxorubicin than with the previous conditions, revealing the influence of the hypoxic conditions of the tumor on drug release.

Analogously, Yu et al. also carried out a release study of doxorubicin, but in this case, it was loaded into 87 nm sized liposomes [54]. First, they evaluated which dialysis membrane was more adequate to determine the permeation kinetics of free doxorubicin. To this end, different types of dialysis membranes were used: regenerated cellulose (RC) membranes with an MWCO of 10 kDa and 20 kDa and cellulose ester (CE) membranes with an MWCO of 8–10 kDa, 20 kDa, 50 kDa, 100 kDa and 300 kDa. For the dialysis membranes of the same type, it was evidenced that the barrier effect of the membranes decreased as the MWCO increased since the drug diffusion rate increased with the increase in the MWCO. For the dialysis membranes of different materials with the same MWCO, CE membranes showed lower doxorubicin diffusion compared with RC membranes. Indeed, CE membranes with 50 kDa exhibited intermediate permeation, whereas RC membranes with an MWCO of 20 kDa showed a faster translocation of doxorubicin in the first 4 h. This difference may have been due to the porosity of the dialysis membranes, CE membranes being less porous than RC membranes, and the interaction between doxorubicin and the membrane materials. Altogether, both MWCO and the membrane material affected the barrier properties of dialysis membranes. Based on this previous validation, two dialysis membranes with rapid and intermediate permeation (RC 20 kDa MWCO and CE 50 kDa, respectively) were selected to study doxorubicin release from liposomes. For this purpose, dialysis tubes were placed into an apparatus that was filled with a release medium composed of 100 mM NH4HCO_3_, 5% sucrose (*w*/*v*), 75 mM MES, 5% 2-hydroxypropyl-beta-cyclodextrin (*w*/*v*) and 0.02% NaN3 (pH 6), and the temperature was set at 45 °C to achieve an accelerated release of doxorubicin. Being aware that when evaluating drug release with dialysis, the membrane permeation process rather than the actual drug release kinetics can determine the rate at which the drug appears in the receptor chamber, these authors developed a mathematical model to calculate the actual drug release kinetics from raw drug release data. The apparent release data systematically underestimated the actual release data with both 20 kDa RC and 50 kDa CE membranes, which may be wrongly attributed to more prolonged drug release from the liposomes. This underestimation was correlated with the barrier effect of the dialysis membrane: the lower the permeability coefficient across the membrane, the bigger the underestimation. Altogether, the authors demonstrated that this could make some membranes unsuitable for the evaluation of drug release kinetics under these conditions because of the fact that the drug release rate from the nanocarrier exceeds the drug permeation rate through the dialysis membranes. This can lead not only to a lower drug concentration in the sampling compartment but also to a violation of sink conditions inside the dialysis bag which, in turn, could limit the rate of drug release from the liposomes.

In the same way, Shen et al. studied the release of oxaliplatin (397.29 Da) from 150 nm sized liposomes [58]. One of these liposomal formulations was prepared by conjugating oxaliplatin prodrug with 1,2-distearoyl-sn-glycero-3-phosphoethanolamine (DSPE) and another one was obtained by conjugating oxaliplatin with dodecanoyl chloride. To test the release profiles of both types of liposomes, they used a dialysis bag (8–14 kDa MWCO), which was immersed in the release media (PBS) in the absence or presence of glutathione, to mimic the tumor environment, and stirred at 200 rpm at room temperature. Both types of liposomes showed a sustained release of oxaliplatin; however, the release from the liposomes incubated in the presence of glutathione was higher (25.99% and 34.23% for each type of liposome) than those incubated in the absence of glutathione (10.67% and 18.85% respectively). So, this method was capable of determining the relationship between the stimulus-dependent release mechanism and glutathione levels in the medium.

#### 3.1.3. Polymeric Nanoparticles

On the one hand, regarding hydrophilic macromolecules, Luo et al. investigated the release of nisin (3354 Da), which was loaded into 112 nm soluble soybean polysaccharide-based nanoparticles [60]. For this purpose, they used a dialysis bag with a 100 kDa MWCO, which was placed in a bath shaker with a rotation speed of 140 rpm at 25 °C. The release medium was acetic acid buffer solution (pH 4) because, at this pH, the nisin interacts with the soybean polysaccharide with a stronger binding force. The authors first validated that nisin in solution freely diffused through the dialysis membrane in the first 3 days so that there was no barrier effect attributable to the dialysis membrane itself. Under these conditions, the release profile of nisin from nanoparticles was extended over 21 days, during which two stages could be defined: in the first 24 h, about 34% of the nisin was released from the nanoparticles, which presumably corresponds to the nisin that was physically adsorbed on the surface of polysaccharides. In the following 20 days, nisin experienced a sustained release, reaching a release of 60% of the total encapsulated nisin, which presumably corresponds to the nisin inside the nanoparticles.

On the other hand, regarding small hydrophilic drugs, Hua et al. prepared 95–445 nm PLGA nanoparticles containing ciprofloxacin (331.3 Da) and magnetite nanoparticles [16]. For the release study, 1 mL of nanoparticle suspension in PBS was placed in a dialysis bag (12–14 kDa MWCO) against 10 mL of PBS used as the release medium. An incubated shaker at 37 °C and 100 rpm was used, and sink conditions were ensured. When no magnetic field was applied, an initial burst release of around 20% of the drug was observed in the first 2 days, followed by a sustained release during the next 4 weeks, achieving a total release of 95% of ciprofloxacin. In addition, ciprofloxacin release from these PLGA magnetic particles could be triggered with an external oscillating magnetic field. This stimulus-responsive release was also assessed with the same dialysis bag method at 20 °C. In this case, a non-physiological temperature was used due to technical limitations in applying the external magnetic field. Notably, PLGA nanoparticles showed an approximately 5-fold higher cumulative release percentage in the presence than in the absence of the external oscillating magnetic field after 6 days. This controllable release feature was also demonstrated in a pulsatile context upon subsequent alternative exposure to the magnetic field.

Abd-Elhalem et al. studied the release of methotrexate (454.43 Da) from two types of nanoparticles: 2–20 nm nano fibrillated nanoparticles (composed of short microfibers of cellulose) and 5–15 nm nano silicon dioxide nanoparticles [64]. The release of methotrexate from these carriers was studied using a dialysis bag with an MWCO of 12–14 kDa, which was placed in PBS pH 7.4 and stirred at 50 rpm. With this dialysis method, the authors were able to study the influence of the formulation parameters on the release profile of the drug. Over a 7-day study, both nanocarriers achieved a sustained release of methotrexate, although the release rate from nano silicon dioxide was slower because the compact structure of these nanoparticles hindered the methotrexate from prompt release.

Simões et al. investigated the release of riboflavin (376.36 Da) and quercetin (302.23 Da) from β-lactoglobulin nanoparticles, which were separately loaded with each compound [65]. They prepared two batches of nanoparticles of different sizes: 200–300 nm and 80–100 nm. The in vitro release profiles of both bioactive compounds were evaluated with dialysis. The dialysis bag (10 kDa) with 5 mL of the sample was placed in a reactor with 50 mL of two food simulant solutions composed of 10 and 50% ethanol (for foods that have a hydrophilic and hydrophobic character, respectively), under continuous stirring at 200 rpm. The release profiles were assessed over 72 h at 4 °C and at 25 °C. When using refrigerated conditions, the results showed a slower release profile of both compounds in comparison with that observed at room temperature, regardless of the food solution, so this dialysis bag technique was able to distinguish release profiles when varying the temperature. However, the temperature-dependent difference between those profiles may simply be due to the loss of sink conditions at 4 °C with reduced solubility of compounds in food simulants rather than to distinct release profiles.

Polymeric nanoparticles of 150 nm containing doxorubicin (543.52 Da) were also developed by Bobde et al. [66]. In these nanoparticles, the drug was conjugated to the polymer backbone with a pH-sensitive hydrazone linker to selectively trigger drug release in an acidic tumor microenvironment. In the in vitro release study, a dialysis bag (3.5 kDa) was used, immersed in 50 mL of PBS at three distinct pH values (i.e., 5.5, 6.5 and 7.4) at 37 °C and shaken constantly at 100 rpm. This method served to evidence the influence of the pH of the release medium on the release profile of doxorubicin, as higher amounts of the drug released in shorter periods of time were achieved with lower pH values.

Regarding small lipophilic molecules encapsulated in polymeric nanoparticles, PLGA-lecithin-PEG core–shell nanoparticles for controlled delivery of docetaxel (807.8 Da) were developed by Chan et al. [67]. To assess drug release, 3 mL of nanoparticles were placed in dialysis microtubes with an MWCO of 10 kDa surrounded by 3 L of distilled water with gentle stirring at room temperature. This technique allowed for the influence of the molecular weight of the polymer on the formulation performance to be determined: the higher the molecular weight, the slower the drug release.

Yang et al. studied the release of curcumin (368.37 Da) from 246 nm poly-L-lysine nanocapsules [69]. In these nanocapsules, the amine group of poly-L-lysine was conjugated at different positions of the polymer chain with three compounds: deoxycholic acid, methoxy polyethylene glycol and cyanine 5.5 for hydrophobic interaction with curcumin, stealth effect, and fluorescent tracking, respectively. An in vitro release study was performed with a dialysis bag with a 20 kDa MWCO immersed into three PBS solutions (pH 5.5, pH 6.8 and pH 7.4) and maintained at 37 °C under continuous stirring at 100 rpm. This method was able to evidence different curcumin release profiles when the pH of the release medium was modified in such a way that as pH decreased, the drug release increased dramatically, resulting in initial burst release. This was due to the fact that poly-L-lysine, which formed the shell of the nanoparticles, became protonated at acid pH. Consequently, the entangled poly-L-lysine chains started to swell due to cationic repulsive forces between chains, resulting in the decrease in the shell density and ultimately, drug release.

Weng et al. prepared different polymeric nanoparticles with an average size of less than 100 nm loaded with itraconazole (705.63 Da), cholecalciferol (384.63 Da) and flurbiprofen (244.26 Da) [47]. To study the release of each drug under sink conditions from all these nanoparticles, a dialysis bag (3.5 kDa MWCO) was immersed into 450 mL of release media (0.1 M HCl for itraconazole; 0.1% SDS *w/v* for cholecalciferol and PBS pH 7.4 for flurbiprofen) at 37 °C under magnetic stirring at 75 rpm. Notably, despite the lower MWCO of the dialysis bag (3.5 kDa) in comparison with the MWCO of the membrane used for centrifugal ultrafiltration (30 kDa) in this same study (as explained in centrifugal ultrafiltration section), dialysis only underestimated drug release in the case of flurbiprofen. In the cases of both itraconazole and cholecalciferol, the percentage of drug released at each timepoint was higher in the dialysis group, which was ascribed to poor compatibility of the dialysis membrane with acidic conditions or nanoparticle destabilization.

#### 3.1.4. Lipid Nanoparticles

Regarding, lipophilic drugs, Aji et al. studied the release of lopinavir (628.8 Da) from 230 nm sized lipid nanoparticles [72]. The assay was performed at pH 1.2 (HCl) for 2 h and at pH 6.8 in PBS for 12 h using a dialysis bag (12 kDa MWCO), and sink conditions were maintained. The dialysis bag was placed into a beaker containing the release medium and was magnetically stirred at 100 rpm. A slow release was shown with both media, with 1.7% of the drug released in HCl during 2 h and 3.6% in PBS at pH 6.8 during 12 h.

Analogously, the release of simvastatin (418.57 Da) from 130 nm sized solid lipid nanoparticles was investigated by Rizvi et al. [74]. They used a dialysis bag (3.5 kDa MWCO) maintained at 37 °C and constantly stirred at 100 rpm, with simulated gastric fluid (SGF, pH 1.2) and simulated intestinal fluid (SIF, pH 6.8) as release media. Moreover, Tween 80 (0.5% *w*/*v*) and ethanol (5% *v*/*v*) were added to maintain sink conditions. With this technique, no differences were observed when the release medium was varied: in the first 2 h, 18% of simvastatin was released in SGF, whereas 13% was released in SIF. In both cases, this initial release was followed by a sustained release pattern with a cumulative release of approximately 50% of simvastatin over 24 h.

Murthy et al. investigated the release of raloxifene hydrochloride (473.58 Da) from 208 nm sized soy lecithin–chitosan hybrid nanoparticles [75]. A dialysis bag (3.5 kDa) immersed in 500 mL of release medium (PBS pH 6.8 with 0.1% *w/v* Tween 80) was used. The system was maintained at 37 °C under continuous stirring at 100 rpm. In the first 4 h, only 25% of raloxifene was released from these nanoparticles, followed by a sustained release with a cumulative release of approximately 70% of raloxifene in 48 h.

#### 3.1.5. Highlights of the Dialysis Bag as Separation Method

Dialysis has the enormous advantage of being able to determine the release of many drugs, preferably of small molecules, with very different characteristics as well as from different nanocarriers with a wide range of sizes, especially from the smallest ones, for which easier techniques are not useful. In the case of microparticles, this is not a common method to study in vitro drug release, since whenever possible, for these larger carriers, simpler techniques like centrifugation can be used to achieve an efficient separation. 

The key point of an efficient separation with this technique is, above all, the selection of a suitable MWCO of the membrane, which must be greater than the molecular weight of the released drug but smaller than the nanocarrier size. This can be a relevant concern in the case of small nanocarriers encapsulating either macromolecules or highly lipophilic drugs that require the addition of surfactants to the release medium to ensure sink conditions. In this latter case, surfactants may form micelles that are retained in the donor compartment if sized above the MWCO threshold, preventing free diffusion of the drug into the receptor medium. In addition, the greater the difference between the molecular weight of the drug and the membrane pore size, the more precise the determination of the release as it will not be limited by the permeation of the drug through the membrane. Additionally, as with most dialysis-based techniques, it cannot be used with drugs that bind to the dialyzing membrane because this will cause clogging of the membrane, which will lead to inefficient separation of the free drug from the encapsulated one and, consequently, to unrealistic release kinetics [77]. This is the reason why it is recommended to assess the suitability of the dialyzing membrane before studying release profiles.

Moreover, the ratio between the volumes of the inner and the outer compartment must be considered, as the volume of the release medium added into the dialysis bag should be at least 6–10-fold less than that of the outer compartment to ensure sink conditions [78].

Furthermore, all these studies mentioned that the determination of drug release was carried out at a constant temperature (usually 37 °C), but, in fact, they did not clarify how the temperature was maintained constant. If the temperature is not maintained constant during the release study, modifications in the release profile can occur.

Apart from the importance of maintaining the temperature constant, the agitation should also be considered. If the study is performed under gentle magnetic stirring on-ly the acceptor compartment can be effectively stirred. If the study is performed under oscillating agitation the entire system is uniformly stirred, although this type of agita-tion is less efficient to remove the unstirred water layer around the membrane. In both cases, the agitation speed does not reach values higher than 200 rpm.

### 3.2. Reverse Dialysis 

Reverse dialysis is a variant of the dialysis technique, in which the position of the acceptor and donor compartments is inverted. In reverse dialysis, the formulation is placed in the outer compartment under gentle agitation. The drug released from the carriers diffuses through the dialysis membrane, with a specific MWCO, into the inner compartment with the release medium. In this case, samples are collected from the small compartment, which acts as the acceptor compartment [79] (Figure 2B). 

Manna et al. investigated the release of bupivacaine, a small (288.43 Da) lipophilic drug, from 23.6 µm multivesicular liposomes, with reverse dialysis [55]. First, they studied the diffusion kinetics of the drug in solution across various MWCO membranes (10 kDa, 20 kDa, 50 kDa and 100 kDa) using the conventional dialysis bag technique. Each dialysis bag was placed into 200 mL of release medium (PBS pH 7) and maintained at 37 °C. It was observed that the diffusion rate of the free bupivacaine increased as the membrane MWCO increased. To minimize any impending effect of the MWCO, the 100 kDa membrane was selected to carry out the reverse dialysis, in accordance with the previous statement by Yu et al. that the membrane must have an MWCO at least 100 times higher than the molecular weight of the drug released. Although the diffusion kinetics is expected to be the same in both directions only if the volumes of the medium are maintained, the authors changed to a reverse dialysis configuration to evaluate the release of bupivacaine from liposomes under the assumption that this change would not have any impact on the diffusion of bupivacaine [54]. Subsequently, using reverse dialysis, the authors studied the release of bupivacaine from liposomes and the effect of three parameters: temperature (25, 31, 37 and 40 °C), agitation speed (120 and 140 rpm) and pH of the release medium (pH 5 and 6 using citrate-phosphate buffer, pH 7 using PBS and pH 10 using carbonate-bicarbonate buffer). The release profile of bupivacaine showed in all cases a three-step pattern: an initial burst release accounted for by the release of bupivacaine from the surface of the multivesicular liposomes, followed by a lag phase, during which no release occurs, ascribed to depletion of the drug on the surface and rearrangement of lipid structure, and a final secondary release phase due to the erosion of the liposomes. Notably, this reverse dialysis technique served to evidence the changes induced in these release profiles by modifying temperature, agitation speed and pH conditions [55].

#### Highlights of Reverse Dialysis as a Dialysis-Based Method for Release Studies 

Reverse dialysis has the advantage of avoiding the formation of immobile water layers and reducing the likelihood of violating sink conditions inside the donor compartment. On the one hand, the avoidance of the formation of an immobile water layer in the donor compartment occurs because stirring can be applied in this compartment. On the other hand, the reduction in the likelihood of violating sink conditions in the donor compartment occurs because this method, in contrast to the normal dialysis method, allows the volume of this compartment to be increased. 

However, because of the strong dilution of the sample in the donor compartment, the concentration gradient to drive drug diffusion through the dialysis membrane toward the acceptor compartment is limited.

### 3.3. Diffusion Cell 

Diffusion cells are other dialysis-based techniques used for testing drug release from nanocarrier formulations. Among them, Franz cells and side-by-side diffusion cells are the most common. Both devices consist of two compartments: a donor compartment, where the formulation is placed, and an acceptor compartment, where the release medium is placed. Both compartments must always be maintained at 32–37 °C to mimic the physiological temperature of either the administration site or the absorption site. Between both compartments, there is a semi-permeable membrane with a specific pore size, which allows the passage of the released drug from the donor to the acceptor compartment [80]. As with normal dialysis, the acceptor compartment is maintained under magnetic stirring to avoid the formation of static water layers and subsequently, the layer of saturated drug around the membrane, which would limit drug diffusion from the donor to the acceptor compartment [25]. 

#### 3.3.1. The Franz Diffusion Cell

In this dialysis cell, both chambers are arranged vertically and separated by a semipermeable membrane. Formulations are placed in the upper compartment (donor) and release the drug through this diffusion barrier into the acceptor medium, from where the samples are taken (Figure 2C). This technique has been applied to study the release from nanoparticles, as shown in Table 4.

##### Polymeric Nanoparticles

On the one hand, regarding hydrophilic macromolecules, Andreani et al. studied the release of insulin (5700 Da) from uncoated and PEG-coated silica nanoparticles [81]. Two different types of PEG coating were used, i.e., PEG 20,000 and PEG 6000. Uncoated, PEG 20,000-coated and PEG 6000-coated nanoparticles had an average size of 289.6, 625.2 and 493.7 nm, respectively. The in vitro drug release assay of insulin was carried out using a Franz diffusion cell with a membrane with an average pore size of 0.2 µm between the donor and acceptor chambers. PBS at pH 6.8 or HCl/KCl buffer at pH 2.0 were used as release media, which were maintained at 37 °C under continuous stirring. This Franz diffusion cell allowed for different release profiles to be evidenced when the nanoparticle coating was modified. Although the average pore size of the membrane is only slightly smaller than the average size of the uncoated nanoparticles, the authors observed that the PEG-coating accelerated the drug release at both pH values in comparison with uncoated nanoparticles since the hydration of the PEG layers was favored, prompting protein release.

On the other hand, the Franz diffusion cell is also a reliable technique to study the release of small molecules such as amikacin (585.6 Da) and moxifloxacin (401.43 Da). Abdelghany et al. studied their release from three types of PLGA nanoparticles: alginate-coated PLGA nanoparticles, with an average size of 640 nm, alginate-loaded PLGA nanoparticles with an average size of 325 nm and PLGA nanoparticles without alginate with an average size of 294 nm [83]. In vitro drug release was determined with a dialysis cell: a nanoparticle suspension was placed in a donor compartment with PBS (pH 7.4 at 37 °C), which was separated from the acceptor compartment by a cellulose membrane (14 kDa MWCO). This acceptor compartment was also filled with PBS (pH 7.4). The volume ratio between the receptor and the donor compartment was fixed at 20. With this dialysis method, differences in the release profiles were observed: alginate PLGA nanoparticles showed a slower release in comparison with PLGA nanoparticles without alginate, whereas no significant differences were found between the alginate-loaded and alginate-coated PLGA nanoparticles. Notably, the distinct size of each type of nanoparticle may also account for the more rapid release rate in the absence of alginate since alginate PLGA nanoparticles had a bigger particle size than those without alginate.

Focusing on lipophilic molecules, Shale et al. studied the in vitro release of dexamethasone (392.46 Da) from various types of pH-sensitive polymeric nanoparticles made from Eudragit ^®^ L 100, Eudragit ^®^ L 100-55, hydroxypropyl methyl cellulose phthalate (HPMCP)-50, HPMCP-55 and cellulose acetate phthalate (CAP) [84]. The in vitro drug release was determined at 32 °C and 600 rpm under non-sink conditions using a Franz diffusion cell, where the acceptor compartment was filled with two different types of buffers: pH 7.5 and pH 4.5 buffer. A membrane of 12–14 kDa MWCO was used. The results showed that the nanoparticles made with the pH-sensitive polymers can control the release profile depending on the pH of the release medium. In fact, if a faster drug release is required at pH 7.5, CAP and HPMCP nanoparticles are the most suitable ones because their swelling, erosion and dissolution kinetics are favored at this pH. However, at pH 4.5 buffer, a slower drug release is achieved with CAP nanoparticles rather than with the HPMCP or Eudragit ones.

###### Lipid Nanoparticles

Regarding lipid nanoparticles, the release of lidocaine (234.17 Da) and prilocaine (220.31 Da), two lipophilic drugs, from nanostructured lipid carriers based on cetyl palmitate and capric/caprylic triglycerides as structural lipids and Pluronic 68 as the colloidal stabilizer was evaluated [86]. The in vitro release study was carried out using a Franz diffusion cell composed of a donor (400 µL) and an acceptor (4 mL) compartment containing 5 mM Tween/PBS (pH 7.4) to provide sink conditions. Both compartments were separated by a dialysis membrane with a 10 kDa MWCO. This system was maintained at 37 °C, with magnetic stirring (300 rpm). A slight initial burst (below 10%) release in the first 30 min was observed, which could be due to the existence of non-encapsulated lidocaine/procaine in the formulations, as the encapsulation efficiency showed values lower than 50% for both anesthetics. Moreover, a nearly complete release of prilocaine and around 80% of lidocaine was achieved over 24 h.

##### 3.3.2. Side-by-Side Cells

Aside-by-side diffusion cell is also composed of two compartments, both filled with release medium and separated by a semipermeable membrane; however, the acceptor and receptor compartment are placed horizontally (Figure 2D). By adding the sample into the donor compartment, the experiment is started, and the released drug diffuses through the membrane into the acceptor compartment, from where sampling occurs [87]. Importantly, in this configuration, both the acceptor and donor chambers can be magnetically stirred independently.

Using these dialysis cells, Sapino et al. investigated the release of methotrexate, a small (454.44 Da) hydrophilic drug, from 200 nm sized mesoporous silica nanoparticles [88]. The nanoparticles were placed in the donor compartment and the release medium (phosphate buffer pH 6.5) was placed in both chambers at 34 ± 1 °C under continuous stirring. The results showed that in the first 5 h, approximately 35% of the entrapped methotrexate was released and, after 24 h, about 70% of the methotrexate was released from these mesoporous silica nanoparticles.

##### 3.3.3. Highlights of Diffusion Cells as Dialysis-Based Methods for Release Studies

Dialysis cells are mostly used to study the release of small-molecule drugs from nanocarrier formulations, not from microcarriers, because for bigger carriers, easier techniques can be used. To study the release of macromolecules from nanocarriers, not many examples utilizing dialysis cells have been reported yet.

In side-by-side diffusion cells, it is important to consider drug solubility in the release medium, as the ratio between acceptor and donor compartment volume is smaller than in the case of Franz diffusion cells, so, to maintain sink conditions with that method, drug solubility should be high. In Franz diffusion cells, as the ratio between the acceptor and the donor compartment volume is bigger than in side-by-side diffusion cells, it is easier to achieve sink conditions in the case of less soluble drugs.

These techniques have the advantage of being developed to simulate diffusion-based transport through biological barriers such as the skin or the gut wall [85,89,90]. Another advantage is that, for side-by-side diffusion cells, as both compartments are magnetically stirred, it is possible to avoid the formation of boundary layers [90]. However, in Franz diffusion cells, only the acceptor compartment can be stirred.

On the other hand, one of the disadvantages of these devices is that, as they also work only through membrane diffusion, membrane pores can be blocked when working with drugs or particles that bind to the membrane, thus reducing the available diffusion surface.

## 4. In Situ Detection Methods

In situ detection methods offer an automation of quality control in drug release experiments [91]. These methods are desirable for the determination of drug release behavior of micro or nanosized carriers as they can measure the in vitro drug release as a function of time, by directly measuring drug concentration in the release medium, so that no drug or formulation is lost during the analysis as it is the case for all the methods mentioned above [92].

### 4.1. The Uv-Vis Detection Method

The UV-Vis detection method is one of the most traditional analytical methods for dissolution testing when the tested compounds exhibit selective absorption in the UV-Vis region. Using this method, it is possible to determine the amount of drug released if there is no analytical overlap in the absorbance region between the signals of the released drug and the constituents of the colloidal systems. However, this technique is restricted by the presence of air bubbles. Moreover, it is restricted to certain molecules as they must be detectable in the UV-Vis region.

Kandile et al. studied the release of curcumin, a small (368.38 Da) lipophilic molecule that exhibits absorption in the UV-Vis region, from 16.8–59.6 nm chitosan, ZnO and gold nanoparticles [93]. The drug release profile was evaluated in acid (pH 5.4) and neutral (pH 7.4) mediums, and the results showed that in acid conditions, the percentage of drug released was higher (23.7–43.98%) than in neutral conditions (10.21–23.84%) in all cases. This could be attributed to the higher swelling of nanoparticles in acid conditions.

This detection method can also be paired with any dialysis-based method to reduce interference with carrier excipients of high molecular weight. For example, in a release study on bupivacaine from multivesicular liposomes, Manna et al. used a dialysis method, and the released drug was in situ detected in the acceptor compartment using in situ fiber optic UV-Vis [55]. Dialysis cells are mostly used to study the release of small molecules.

### 4.2. The Fluorescence Detection Method

Alternatively, drug release behavior can be quantified with fluorescence, which has higher sensitivity than UV-Vis detection and is less prone to interferences with other substances in the medium. As only a few molecules naturally fluoresce, this technique is only implementable for a small number of drugs. Dai et al. studied the release of doxorubicin, a small (543.52 Da) hydrophilic drug, from 95 nm sized gadolinium chelate-conjugated temperature-sensitive liposomes [94]. The in vitro doxorubicin release was determined following the drug fluorescence in 2 mL of water with or without 808 nm laser irradiation. The results showed a doxorubicin release in the first 100 min that was five times higher in the presence of laser irradiation (55%) than without it (10%). In the same way, Zhao et al. also studied the release of doxorubicin from 92 nm sized peptide-modified pH-sensitive liposomes [51]. Their study showed that the pH-sensitive liposomes were unstable at acid pH, which could lead to the encapsulated drug being released from the liposomes in these conditions. To carry out the release measurements, they added an aliquot of liposomes into 2 mL of PBS solution (pH 5.5; 6.0, 6.5 or 7.4) in a quartz cell at 37 °C, and the fluorescence intensity of the solution was monitored using a spectrofluorometer. These release experiments confirmed that in the first 24 h, the release of doxorubicin in acid conditions was five times higher than that achieved in neutral ones.

### 4.3. Other in Situ Detection Methods

Other in situ detection methods such as Raman and infrared (IR) spectroscopy can be used to assess in vitro drug release. Raman spectroscopy is a nondestructive technique that involves illuminating a sample with monochromatic laser light and detecting what scatters back. Schaefer et al. utilized this technique to monitor the release of a model drug, 3-nitrobenzene sulfonate, from 1 µm sized liposomes [95]. Regarding IR, it is an absorption method that detects the interaction between IR radiation and drugs by absorption/emission or reflection. Ghosh et al. investigated the release of ciprofloxacin from 100 nm sized liposomes. As this drug is photoactivable, its release can be measured in the IR spectra [96]. Both techniques require high drug concentrations to provide reliable results, which is usually difficult to achieve due to the poor solubility of the drug in the release medium. Moreover, the instruments are complex and expensive. Furthermore, in IR spectroscopy, aqueous release studies cannot be carried out as water causes significant interferences because it has two important infrared absorption peaks.

### 4.4. Highlights of In Situ Detection Methods for Release Studies

The aforementioned in situ detection methods have the great advantage of being automatic since online measurements are carried out, which leads to a reduction in experimental errors. Moreover, a direct measure of the released drug in the medium can be carried out with no need for further processing steps, which means the free drug can be measured in the presence of the drug loaded in the carriers.

One of the disadvantages of these methods is that the validation procedures can be complex and not cost-efficient. Moreover, these methods are limited to a certain number of molecules since they require drugs to have specific characteristics to be detected (i.e., fluorophore or chromophore groups).

## 5. Outlook

In contrast to macro-dosage forms, colloidal carriers lack standardized procedures to determine their in vitro drug release profiles. However, standardized in vitro release studies are needed as a subrogate of in vivo release kinetics for both formulation development and quality control at the industrial level to evaluate variability between batches. With no compendial or regulatory standard and with the variety of testing methods described in the literature, direct comparison among the release profiles from different colloidal systems is currently difficult [97].

The major challenge is to find the most adequate in vitro release testing method for each colloidal system. To this end, the physical and chemical properties of both the drug delivery system (i.e., particle size and carrier composition) and the encapsulated drug (i.e., molecular weight and water solubility) must be considered [98] (Table 5).

This lack of standardized protocols is mostly due to the difficulties encountered in achieving an efficient, reproducible and rapid separation of the free drug from the encapsulated one due to the small particle size of drug delivery systems, which are not encountered in the case of macro-sized dosage forms.

In this context, for microparticles, due to their bigger size, it is often easier to separate the released drug from the microcarrier by simply using centrifugation with relatively low centrifugal force and time in comparison with those used for nanocarriers [33]. These gentle conditions allow the structure of the microcarrier to be kept unaltered. Different parameters may be adjusted when assessing drug release from these carriers such as the sample volume (which ranged from 0.5 to 1.5 mL), release medium and centrifugation force (2000–40,000× *g*) and time (15 min–1 h). Alternatively, dialysis-based techniques have occasionally been used for release studies from microparticles [56].

In the case of nanocarriers, different techniques have been reported to determine drug release from liposomes or nanoparticles, including ultracentrifugation with high centrifugal forces over long periods of time [33,34,35], centrifugal ultrafiltration [41] or dialysis. When ultracentrifugation is used, the high centrifugal force can lead to alterations in the nanocarriers and lead to an artificial increase in drug release. Alternatively, when centrifugal ultrafiltration is used, the centrifugal force and time are reduced in comparison with ultracentrifugation because, in this case, the centrifugation force is supplemented with filtration through a semipermeable membrane. However, dialysis-based methods such as dialysis bags and diffusion cells are the most common techniques used to determine drug release from nanocarriers [58,61,64,68,72,82,83,87]. The MWCO of semipermeable membranes must be smaller than the MWCO used when testing drug release from microparticles to ensure efficient separation of the nanocarriers from the released drug.

In these cases, the molecular weight of the drug substance is also important since the separation between the released and the encapsulated drug is less efficient when the particle size of the nanocarrier and the molecular weight of the drug are similar. Most release methods are suitable for small molecules by simply utilizing either an MWCO that allows the passage of the free drug, in the case of membrane-based methods [41,44,46,49,57,59,64,68,72,83,85,87], or a packing material that allows the free drug to elute, in the case of SEC [50]. However, to study the in vitro release of macromolecules above 5 kDa, only centrifugation has been reported as the release method [33,36,38] since it achieves the most efficient separation in those cases where the molecular weight of the drug substance approaches the particle size of the nanocarrier.

The aqueous solubility of the drug substance must also be considered to maintain sink conditions during a release study to enable the straightforward release of the drug from the carrier. Otherwise, the drug might prematurely saturate the medium, and the release profile would then be biased in the sense that it would be controlled by the solvent replacement frequency (in the case that the whole medium is centrifuged) or sample aliquot replacement (in the case that aliquots are withdrawn at each sample time). This is especially relevant because many of these colloidal systems are used to deliver poorly water-soluble drugs. Although all sample and separate methods are compatible with the volume of release medium needed for the maintenance of sink conditions even for less water-soluble drugs, among dialysis-based methods, dialysis bags seem to be better suited for this purpose than diffusion cells. This is because in a dialysis bag the volume of the acceptor compartment can be much greater than the volume of the donor compartment in comparison with diffusion cells (particularly in their side-by-side configuration), where the volume ratio between the acceptor compartment and the donor compartment is much lower. Therefore, among dialysis-based methods, the stirring speed used in diffusion cells is higher than in dialysis bags due to the smaller volume of the acceptor compartment in diffusion cells, which makes the formation of stagnant water layers more likely [99]. Moreover, centrifugation is also more compatible with a wider variety of release media to ensure sink conditions than release methods that utilize a semi-permeable membrane, like centrifugal ultrafiltration or dialysis-based methods, where the compatibility of all constituents of the release medium must ensure physical and chemical compatibility with the membrane composition. Despite all these considerations, many studies do not provide information on whether sink conditions are ensured.

In this context, there is a need to validate the release method to be used to ensure that it is reliable enough to discern between the released and the encapsulated drug, although this validation is routinely reported in only a small percentage of the studies [23,35,45,46,69]. Centrifugation force and time were validated by Cai et al., who studied the influence of different relative centrifugation forces and times to achieve the most efficient separation of the free drug from the encapsulated one [36]. However, many authors reported the centrifugation speed (rpm) rather than the relative centrifugation force (g), which does not allow for a straightforward comparison among studies to be drawn because the relative centrifugation force relies on the radius of the centrifugation rotor. In vitro release methods, which include the use of a semi-permeable membrane, also require membrane validation, similar to Yu et al.’s study exploring the influence of the different compositions of a membrane as well as the influence of the pore size in the diffusion of the free drug [54]. However, most of the studies do not make a previous validation, which can lead to errors in subsequent release experiments. In fact, despite having established that the dialysis membrane MWCO should be at least 100 times higher than the molecular weight of the drug, not all studies meet this requirement, and thus, an efficient separation between the released drug and the encapsulated one cannot be ensured [58].

The release method should also serve to study release profiles when different parameters such as the size or the composition of the carrier, the conditions of the release medium, etc., are varied. For example, using centrifugation as a sample and separate method, Dutta et al. were able to demonstrate that the BSA release profile varied with the particle size of PLGA nanoparticles [37]. Analogously, Abd-Elhalem used the dialysis bag method to evidence the role played by the carrier composition in the release of methotrexate [64] and Abdelghany et al. used the Franz diffusion cell technique to study the release of amikacin and moxifloxacin [83]. Sometimes, the influence of the pH or the ionic strength of the release medium can also alter the release profile, and the technique used to determine in vitro drug release must serve to study the influence of these factors on drug release [30,37,66].

Colloidal carriers are often developed to increase the therapeutic index of drug substances, mostly with targeted delivery using stimuli-responsive carriers [100]. In this sense, endogenous factors such as the pH or the ionic strength of the release medium or the presence of redox agents (like glutathione) in the release medium have been demonstrated to trigger drug release. These endogenous factors can be modified in some techniques such as centrifugation/ultracentrifugation, whereas special care must be taken in those techniques that use a membrane since these factors can alter its integrity. Drug release can also be controlled using thermo-responsive systems, which should retain their drug loaded at body temperature (37 °C) and deliver the drug upon local moderate heating (40–42 °C), as occurs in some tumors [101]. Sample and separate methods (including membrane-based methods) are compatible to evaluate drug release from carriers exploiting a thermo-responsive release mechanism since in no case are they directly exposed to the temperatures of the release medium. However, in dialysis-based methods, the integrity of the semipermeable membrane over the whole range of temperatures must be ensured to efficiently study drug release from thermo-responsive carriers.

Although there is currently no compendial or regulatory standard on which is the most suitable technique to determine drug release from each colloidal drug delivery system, the trend observed so far in the literature indicates a preference for sample and separate methods. These methods are preferred for being more simple, more compatible with sink conditions and more representative of the in vivo situation by not artificially interposing a membrane that separates the free drug from the encapsulated one during the implementation of a release study. However, in those cases in which it is ensured that the permeation across an interposed membrane does not represent the limitation factor of the release process, dialysis-based techniques are preferred, given that they do not require any separation procedure subsequent to sampling.

## Figures and Tables

**Table 1:** Examples of colloidal carriers in which in vitro drug release has been evaluated using centrifugation/ultracentrifugation. RCF: relative centrifugal force; PLGA: poly (lactic-co-glycolic acid); SPC: soybean phospholipid; HSPC: hydrogenated soybean phospholipid; TTP: tripolyphosphate; PBS: phosphate-buffered saline; NaCl: sodium chloride.

Colloidal System	Drug Molecular Weight	Drug Solubility	Drug	Carrier Description	RCF and Time	Release Medium	Reference
Microparticles	Macromolecule	Amphiphilic	Interferon-alpha	10 µm sized PLGA microparticles	2000× *g*; 15 min	PBS (pH 7.4) + 0.02% (*w*/*v*) Tween 80	[33]
				10–40 µm sized PLGA/poloxamer microparticles			
	Small molecule	Hydrophilic	Ciprofloxacin	10 µm sized PLGA microparticles	41,400× *g*; 30 min	PBS (pH 7.4)	[29]
			Trimethoprim	1–9 µm sized PLGA 503H microparticles	20,817× *g*; 15 min	Artificial urine (pH 5)	[34]
				1–9 µm sized PLGA 2300 microparticles			
Liposomes	Small molecule	Lipophilic	Curcumin	84 nm sized SPC liposomes	15,000× *g*; 15 min	Saliva, gastric and intestinal fluid	[35]
				93 nm sized SPC: HSPC (7:3) liposomes			
				183 nm sized SPC: HSPC (5:5) liposomes			
				220 nm sized SPC: HSPC (3:7) liposomes			
				146 nm sized HSPC liposomes			
Polymeric nanoparticles	Macromolecule	Hydrophilic	Interferon-alpha	280 nm sized PLGA/Poloxamer nanoparticles	22,000× *g*; 15 min	PBS (pH 7.4) + 0.02% (*w*/*v*) Tween 80	[33]
			Bovine serum albumin	40–1000 nm sized chitosan/TTP nanoparticles	21,000 to 300,000× *g*; 30–90 min	5 wt% trehalose solutions + NaCl	[36]
			Bovine serum albumin	250–<1000 nm sized PLGA nanoparticles	14,000× *g*; 15 min	PBS + Tween 80	[37]
	Small molecule	Hydrophilic	Azelaic acid	295 nm sized PLGA nanoparticles	40,000× *g*; 30 min	PBS (pH 7.4)	[28]
			Ciprofloxacin	300 nm sized PLGA nanoparticles	41,400× *g*; 30 min	PBS (pH 7.4)	[29]
			Trimethoprim	200–400 nm sized PLGA nanoparticles	28,817× *g*; 30 min	Artificial urine (pH 5)	[34]
		Lipophilic	Paclitaxel	161 nm sized PLGA nanoparticles	10,000 rpm; 10 min	PBS/PBS +0.2%Tween 80/PBS + 50% FBS	[38]

**Table 2:** Examples of colloidal carriers in which in vitro drug release has been evaluated using centrifugal ultrafiltration. RCF: relative centrifugal force; PEG: polyethylene glycol; PBCA: poly (butyl cyanoacrylate); HEPES: 2-[4-(2-hydroxyethyl) piperazin-1-yl] ethanesulfonic acid.

Colloidal System	Drug Molecular Weight	Drug Solubility	Drug	Carrier Description	RCF and Time	Membrane MWCO	Release Medium	Reference
Liposomes	Small molecule	Hydrophilic	Topotecan	100 nm sized pegylated liposomes	14,000 rpm; 10 min	30,000 Da	pH 5.10/pH 3.35–4.10	[23]
			Ciprofloxacin	80–90 nm sized unilamellar vesicles	8100× *g*; 10 min	10,000/30,000 Da	HEPES-buffered saline	[41,42]
Micelles	Small molecule	Hydrophilic	Doxorubicin	50–100 nm sized PEG micellar formulations	14,000 rpm; 10 min	10,000 Da	Buffer solution with pH 5.0 and 7.4	[43]
Polymeric nanoparticles	Small molecule	Hydrophilic	Primaquine	150–200 nm sized PEG nanoparticles	1000× *g*; 5 min	3 kDa	PBS (pH 7.4)	[44]
			Methotrexate	218 ± 6 nm sized PLGA nanoparticles	2095× *g*; 5 min	50 kDa	Water (pH 5.5)/phosphate buffer (pH 5)	[45]
			Moxifloxacin	418 ± 90.2 nm sized PBCA nanoparticles	10,000× *g*; 20 min	30 kDa	PBS pH 7.4	[46]
		Lipophilic	Itraconazole	100 nm sized d-α-tocopheryl polyethylene glycol 1000 succinate nanoparticles	1000× *g*; 5 min	30 kDa	0.1 M HCl	[47]
			Cholecalciferol	100 nm sized-α-tocopheryl polyethylene glycol 1000 succinate nanoparticles	1000× *g*; 5 min	30 kDa	0.1% *w/v* sodium dodecyl sulphate	
			Flurbiprofen	100 nm sized d-α-tocopheryl polyethylene glycol 1000 succinate nanoparticles	1000× *g*; 5 min	30 kDa	PBS pH 7.4	
Lipid nanoparticles	Small molecule	Hydrophilic	Dibucaine	200 nm sized lipid nanoparticles	4100× *g*; 20 min	-	PBS (pH 7.5)	[48]
			Methotrexate	211 nm sized lipid nanoparticles	2095× *g*; 15 min	50 kDa	Water pH 5.5/PBS (pH 5)	[45]

**Table 3:** Examples of colloidal carriers in which in vitro drug release has been evaluated using the dialysis bag method. PLGA: poly (lactic-co-glycolic acid); PEG: polyethylene glycol; GSH: glutathione; FBS: fetal bovine serum; NH4HCO_3_: ammonium hydrogen carbonate; MES: 2-(N-morpholino) ethanesulfonic acid; HP-CD: 2-Hydroxypropyl-beta-cyclodextrin; NaN3: sodium azide; PBS: phosphate buffered saline; HCl: hydrogen chloride; SDS: sodium dodecyl sulfate.

Colloidal System	Drug Molecular Weight	Drug Solubility	Drug	Carrier Description	Agitation Speed	Membrane MWCO	Release Medium	Reference
Microparticles	Small molecule	Hydrophilic	Ciprofloxacin	0.5–6 µm sized PLGA microparticles	100 rpm	12–14 kDa	PBS	[56]
Liposomes	Small molecule	Hydrophilic	Doxorubicin	165 nm sized pegylated liposomes	Undisclosed	10 kDa	PBS (pH 7.4) + GSH//FBS	[57]
			Doxorubicin	87 nm sized liposomes	Undisclosed	20 and 50 kDa	100 mM NH_4_HCO_3_ + 5% sucrose (*w*/*v*) + 75 mM MES + 5% HP-CD (*w*/*v*) + 0.02% NaN_3_ (pH 6)	[54]
			Oxaliplatin	150 nm sized liposomes	200 rpm	8–14 kDa	PBS//PBS+GSH	[58]
			Platinum	150 nm sized light activable liposomes	100 rpm	3.5 kDa	PBS	[59]
Polymeric nanoparticles	Macromolecule	Hydrophilic	Nisin	112 nm sized soluble soybean polysaccharide-based nanoparticles	140 rpm	100 kDa	Acetic acid buffer solution (pH 4)	[60]
			Exenatide	200 nm sized PEGylated reverse micelle-loaded lipid nanocapsules	Undisclosed	100 kDa	Fasted state-simulated gastric fluid and fasted state-simulated intestinal fluid	[61]
			Insulin	95–200 nm sized anionic polyelectrolyte nanoparticles complexes	700 rpm	1000 kDa	Fasted state small intestinal fluid + 0.001% (*w*/*v*) of methylcellulose	[62]
	Small molecule	Hydrophilic	Ciprofloxacin	95–200 nm sized PLGA nanoparticles	100 rpm	12–14 kDa	PBS	[56]
			Zidovudine	432 nm sized glutamic acid–alginate nanoparticles	Undisclosed	14 kDa	PBS (pH 7.4)	[63]
			Methotrexate	2–20 nm sized fibrillated nanoparticles and 5–15 nm sized silicon dioxide nanoparticles	50 rpm	12–14 kDa	PBS (pH 7.4)	[64]
			Riboflavin	100 and 200–300 nm sized β-lactoglobulin nanoparticles	200 rpm	10 kDa	Hydrophilic and hydrophobic food solutions	[65]
			Quercetin					
			Doxorubicin	150 nm sized polymeric nanoparticles	100 rpm	3500 Da	PBS (pH 5.5, 6.5 and 7.4)	[66]
		Lipophilic	Docetaxel	100 nm sized PLGA-lecithin-PEG core–shell nanoparticles	Undisclosed	10 kDa	Distilled water	[67]
			Sorafenib	240 nm sized polymeric nanoparticles	100 rpm	12–14 kDa	PBS (pH 7.4) + 1% of Tween 80	[68]
			Curcumin	246 nm sized cored poly-L-lysine nanoparticles	100 rpm	20 kDa	PBS (pH 5.5, 6.8 and 7.4)	[69]
			Rifampicin	260.3 nm sized N-2-hydroxypropyl methacrylamide co-polymer-PLGA nanoparticles	120 rpm	5 kDa	PBS (pH 7.4)	[70]
			Itraconazole	100 nm size d-α-tocopheryl polyethylene glycol 1000 succinate nanoparticles	75 rpm	3.5 kDa	0.1 M HCl	[47]
			Cholecalciferol	100 nm sized d-α-tocopheryl polyethylene glycol 1000 succinate nanoparticles	75 rpm	3.5 kDa	0.1% SDS *w*/*v*	[47]
			Flurbiprofen	100 nm sized d-α-tocopheryl polyethylene glycol 1000 succinate nanoparticles	75 rpm	3.5 kDa	PBS (pH 7.4)	[47]
Lipid nanoparticles	Small molecule	Hydrophilic	Phenylethyl resorcinol	218 nm sized lipid nanoparticles	50 rpm	8–14 kDa	Saline media	[71]
		Lipophilic	Lopinavir	230 nm sized lipid nanoparticles	100 rpm	12 kDa	PBS (pH 6.8)//HCl (pH 1.2)	[72]
			Dexamethasone	Core–multishell nanocarriers	100 rpm	3.5 kDa	PBS	[73]
			Simvastatin	130 nm sized solid lipid nanoparticles	100 rpm	3.5 kDa	Simulated gastric fluid (pH 1.2)//simulated intestinal fluid (pH 6.8)	[74]
			Raloxifene hydrochloride	208 nm sized soy lecithin–chitosan hybrid nanoparticles	100 pm	3.5 kDa	PBS (pH6) + 0.1% *w/v* Tween 80	[75]
			Clotrimazole	275 nm sized solid lipid nanoparticles	100 rpm	12–14 kDa	PBS (pH 7.4) + 1% Tween 0	[76]

**Table 4:** Examples of colloidal carriers in which in vitro drug release has been evaluated using Franz diffusion cells. PLGA: poly (lactic-co-glycolic acid); PEG: polyethylene glycol; HPMCP: hydroxypropyl methyl cellulose phthalate; CAP: cellulose acetate phthalate; HCl: hydrogen chloride; KCl: potassium chloride; NaCl: sodium chloride; Na_2_HPO_4_: disodium phosphate.

Colloidal System	Drug Molecular Weight	Drug Solubility	Drug	Carrier Description	Agitation Speed	Membrane MWCO	Release Medium	Reference
Polymeric nanoparticles	Macromolecule	Hydrophilic	Insulin	500–600 nm sized PEG-coated and 300 nm sized uncoated silica nanoparticles	Undisclosed	0.2 µm	PBS (pH 6.0)//HCl/KCl (pH 2)	[81]
	Small	Hydrophilic	Acetazolamide	200 nm sized Eudragit^®^ and 100 nm sized ethylcellulose nanoparticles	200 rpm	12 kDa	NaCl + Na_2_HPO_4_ + NaH_2_PO_4_ (pH 7.2)	[82]
			Amikacin	640 nm sized alginate coated PLGA nanoparticles	Undisclosed	14 kDa	PBS (pH 7.4)	[83]
				325 nm sized alginate loaded PLGA nanoparticles				
				294 nm sized non PLGA modified nanoparticles				
			Moxifloxacin	640 nm sized alginate coated PLGA nanoparticles	Undisclosed	14 kDa	PBS (pH 7.4)	[83]
				325 nm sized alginate loaded PLGA nanoparticles				
				294 nm sized non-PLGA-modified nanoparticles				
		Lipophilic	Dexamethasone	233.7 nm sized Eudragit^®^ L 100-55 nanoparticles	600 rpm	12–14 kDa	Buffer (pH 7.5 and 5.5)	[84]
				250.6 nm sized Eudragit^®^ L 100-55: Eudragit^®^ L100 (1:1) nanoparticles				
				260.8 nm sized HPMCP-50: HPMC-55 (1:1) nanoparticles				
				263.6 nm sized CAP nanoparticles				
		Amphipathic	Melatonin	150–180 nm sized ethylcellulose nanocapsules	Undisclosed	12 kDa	PBS (pH 7.4)	[85]
Lipid nanoparticles	Small	Hydrophilic	Topotecan	108–168 nm sized lipid nanocapsules	300 rpm	12 kDa	Acetate buffer (pH 4.5)	[49]
		Lipophilic	Lidocaine	276–286 nm sized Cetyl palmitate + capric/caprylic triglycerides + Pluronic 68 lipid nanocapsules	300 rpm	10 kDa	5 mM Tween/PBS (pH 7.4)	[86]
			Prilocaine	276–286 nm sized Cetyl palmitate + capric/caprylic triglycerides + Pluronic 68 lipid nanocapsules	300 rpm	10 kDa	5 mM Tween/PBS (pH 7.4)	[86]

**Table 5:** Comparison of all the different testing methods for drug release from colloidal vehicles regarding which type of carrier and which type of drug are most suitable, key parameters for each technique and their advantages and disadvantages. RCF: relative centrifugal force.

	Type of Carrier	Type of Drug	Key Parameters	Advantages	Disadvantages
**Centrifugation/** **ultracentrifugation**	Microcarrier Nanocarrier	Macromolecule Small molecule	RCF Time	Low resource consuming	Not suitable for early sampling points Particle damage
**Centrifugal** **ultrafiltration**	Nanocarrier	Small molecule	Membrane MWCO RCF Time	Lower RCF and time No particle damage	Membrane clogging
**Size exclusion** **chromatography**	Nanocarrier	Macromolecule Small molecule	Packaging pore size	No particle damage	Particle adsorption
**Dialysis bag**	Nanocarrier	Macromolecule Small molecule	Membrane MWCO Temperature Agitation Compartment volume ratio	Versatile Easily compatible with in situ detection methods	Membrane clogging
**Reverse dialysis**	Nanocarrier	Small molecule	Membrane MWCO Temperature Agitation Compartment volume ratio	Avoidance of immobile water layers in the donor compartment Reduce violation of sink conditions in the donor compartment	Membrane clogging
**Diffusion cell**	Nanocarrier	Small molecule	Membrane MWCO Temperature Agitation Compartment volume ratio	Compatible with biological barriers	Membrane clogging
**UV-Vis** **Fluorescence** **Other in situ detection methods**	Microcarrier Nanocarrier	Macromolecule Small molecule	UV-Vis absorption/fluorescence/light scattering/IR absorbance Temperature Agitation	Measure instantaneous release	Limited to certain molecules with specific absorption/emission features

## Data Availability

Not applicable.
